# The Management of Foreign Body Displacement into the Maxillary Sinus as a Complication of Maxillofacial Interventions: Systematic Review

**DOI:** 10.1007/s12070-020-02153-9

**Published:** 2020-09-18

**Authors:** Bartosz Wojtera, Angelika Woźna, Oskar Komisarek

**Affiliations:** 1 Students Research Group of Maxillofacial Orthopaedics and Orthodontics, Poznan University of Medical Sciences, ul. Bukowska 70, 60-812 Poznan, Poland; 2 Department of Maxillofacial Orthopaedics and Orthodontics, Poznan University of Medical Sciences, ul. Bukowska 70, 60-812 Poznan, Poland

**Keywords:** Foreign body, Maxillary sinus, Complications, Implants

## Abstract

Displacement of foreign bodies into the maxillary sinus shows an increasing tendency, especially in regard to raising amount of dental implant installation procedures. The purpose of our study was to compare the efficiency and the rate of late complications among the methods of removal of foreign bodies from maxillary sinus. We performed a systematic review following PRISMA Checklist, searching Pubmed and Google Scholar databases for studies investigating the methods of removal of foreign bodies from maxillary sinus. The inclusion criteria embraced the examined group of at least 10 cases and the follow up period of minimum 3 months. We qualified 7 papers from 531 identified in primary search. Among qualified studies functional endoscopic sinus surgery used in order to remove foreign body from maxillary sinus had no late complications, whereas they occurred in 0–5% cases of using replaceable or pedicled bone approaches and in 15–18% cases of Caldwell-Luc approach. FESS probably should become a gold standard in retrieving foreign bodies from maxillary sinus, however poor evidence requires further investigation, especially in prospective, randomized trials.

## Introduction

The anatomic conditions of maxillary sinus carry the risk of complications, when medical procedures are performed in this region. Iatrogenic displacement of foreign body into the maxillary sinus shows increasing tendency, especially in regard to raising amount of dental implant installation procedures and their further accidental migration [1]. Other types of objects dislocated into the sinus during medical procedures include fractured teeth roots [2], whole teeth [3], endodontic materials and tools [4, 5], dental filling materials [6, 7], dental burs [8], or dental impression materials [9]. Not only dental procedures might induce a foreign body into the sinus—the complication can be caused by otorhinolaryngologists by accidental leaving of gauzes used to nasal or sinus packing [5], or other materials [10].

Removal of the displaced foreign body might cause major difficulties regarding a method of management and ability to perform certain procedures. There is a deficiency of reliable guidelines how to treat the displaced object [11].

The aim of the study was to compare efficiency and late complications related to foreign body removal procedures reported in literature.

## Methods

The systematic review was performed following the PRISMA checklist—Preferred Reporting Items for Systematic Reviews and Meta-Analyses [12]. There is no existing study protocol indicating the justification, hypothesis nor specific methods. The review is not registered in the Prospective International Register of Systematic Reviews (PROSPERO), due to not meeting registration criteria.

The systematic review followed PICO Framework: P (population) refers to patients with foreign body displaced into the maxillary sinus, as the complication of interventions in the maxillofacial region; I (intervention) and C (comparison) referring to the certain methods of removal of foreign body; and O (outcome) refers to the possible late complications in the certain methods of removal follow-up.

The inclusion criteria concerned original studies referred to removal of maxillary sinus displaced foreign bodies with at least 10 cases described in the article and the minimum follow-up period of 3 months.

All studies, which had not met the inclusion criteria were rejected, as well as reviews, single case reports, letters to the editors and chapters of books. We omitted all non-English language articles.

We performed a research on PubMed and Google Scholar databases in using following keywords: *foreign body, root, tooth, implant, endodontic material, retrieval, removal, displacement, maxillary sinus, paranasal sinus, antrum* and their plural forms in titles and abstracts. Afterwards, search results were put in the chronological order to eliminate duplicates.

Titles and abstracts were screened by two authors. Full texts were obtained if they were needed to decide whether to qualify the piece. Afterwards, authors obtained full texts of all included studies to analyze them in detail.

Comparative table based on included studies was constructed. The extracted data was comprised of: the type and the number of treated foreign body displacement, the methods of treatment, the late complications and the period of follow-up (Table 1).

**Table 1:** Qualified studies detailed characteristics

Title	Author, country	Year	Type of Foreign Body (FB)	Method of removal	No. of FB	Late complications	Type of complications	Follow up (months)
Retrieval of Root Fragment in (…) [2]	Hu et al. Egypt, China	2015	Fractured Roots	Replaceable bone lid	21	5% (n = 1)	Nasal discharge and feeling of fullness	3–36
The management of complications of following displacement (…) [15]	Chiapasco et al. Italy	2009	Dental implants	Nasal endoscopy	6	–	–	24
				Replaceable bone lidor pedicled bone lid	17	6% (n = 1)	Maxillary sinusitis 2 years after procedure	
				Nasal Endoscopy + communication closure	4	–	–	
Complications and management of implant migrated (…) [16]	Manor et al. Israel	2018	Dental implants	Caldwell-Luc	52	15% (n = 8)	Maxillary sinusitis and/or oroantral fistula – –	> 12
				Nasal endoscopy	1	–		
				Nasal endoscopy + Caldwell Luc	1	–		
				Spontaneous	1	–	–	
Displacement of dental implants into (…) [1]	Sgaramella et al. Italy	2016	Dental implants	Replaceable bone lid or Calwell-Luc	21	–	–	12
Transnasal endoscopic removal of (…) [14]	Matti et al. Italy	2013	Dental implants	Nasal endoscopy	16	–	–	3–96
The 'dobule-barrel' approach to the (…) [17]	Albu Romania	2013	Dental implants	The double barrel approach or canine fossa trocar approach	50	18% (n = 9)	Maxillary sinusitis	12
Displaced dental materials in the maxillary sinus (…) [11]	Brescia et al. Italy	2019	Dental implants (5), fractured roots (2), dental fillings (2), bone graft, dental implant and dental bur	Replaceable bone lid or pedicled bone lid	2	–	–	3
				Nasal endoscopy	5	–	–	
				Nasal endoscopy + replaceable bone lid	2	–	–	
				Nasal combined endoscopy	2	–	–	

Included studies underwent risk of bias assessment. Two authors independently analysed the contents. The quality of studies was evaluated following the criteria proposed by Cericato et al. [13] by both authors independently. The appraisal estimated as: high quality (10–12 points), moderate quality (6–9 points) and poor quality (5 and less points). Any inconsistency between the authors was resolved by discussion.

Summing up, we compared contents of selected studies using forms of narrative and tabular comparison to obtain review of treatment methods of foreign body displaced into the maxillary sinus as a complication of maxillofacial interventions.

## Results

**Fig.1:**
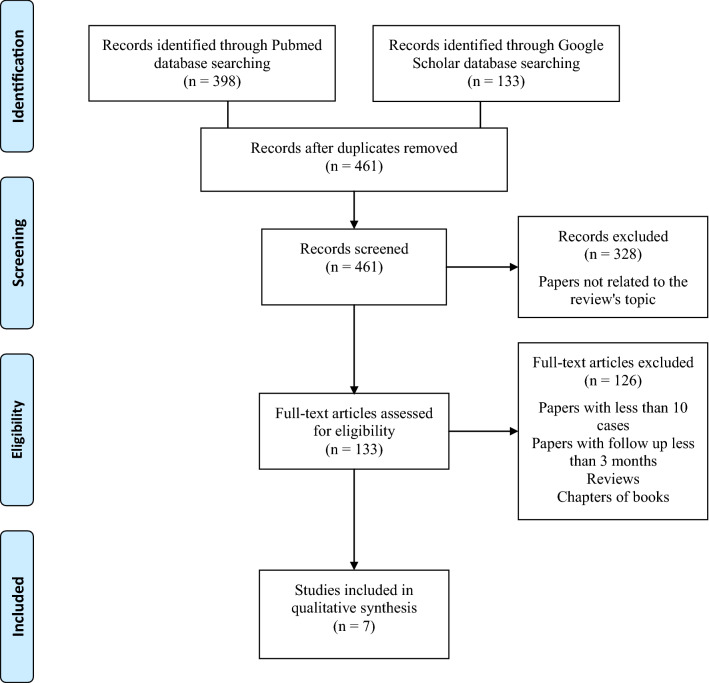
The flow diagram—evidence search and selection

Our search revealed 398 articles on Pubmed database and 133 articles on Google Scholar database. First we screened Pubmed findings, resulting in rejection of 265 papers, which were not related to the study's concern. Another 126 articles were rejected due to not meeting inclusion criteria. 7 papers were qualified to analysis. Afterwards we screened Google Scholar findings, resulting in rejection of: 33 papers which were not related to our topic, 35 papers which were doubled with Pubmed findings, and 65 due to not meeting inclusion criteria. 2 papers, which were doubled with Pubmed findings, were qualified to the analysis. Summary available in the Flow Diagram (Fig. 1).

Qualified studies detailed characteristics are presented in Table 1. Assessment of risk of bias within the studies is presented in Table 2.

**Table 2:** Quality assesment of qualified studies

Title	Author	Q.1 1 point	Q.2 1 point	Q.3 3 points	Q.4 1 point	Q.5 2 points	Q.6 2 points	Q.7 1 point	Q.8 1 point	Amount 12 points	Quality
Retrieval of root fragment in maxillary sinus via anterolateral wall of the sinus to preserve alveolar bone [2]	Hu et al.	+	+	−	−	+ +	−	+	−	5	Poor
The management of complications following displacement of oral implants in the paranasal sinuses: (…) [15]	Chiapasco et al.	+	+	−	−	+ +	−	+	−	5	Poor
Complications and Management of Implants Migrated into the Maxillary Sinus [16]	Manor et al	+	+	+	−	+ +	+	+	−	7	Moderate
Displacement of Dental Implants Into the Maxillary Sinus: A Retrospective Study of Twenty-One Patients [1]	Sgaramella et al.	+	−	+	−	+ +	−	+	−	5	Poor
Transnasal endoscopic removal of dental implants from the maxillary sinus [14]	Matti E. et al	+	+	+ +	−	+	−	+	−	6	Moderate
The 'double-barrel' approach to the removal of dental implants from the maxillary sinus [17]	Albu et al.	−	+	+ + +	+	+ +	+ +	+	−	10	High
Displaced Dental Materials in the Maxillary Sinus: An Original Series Analysis and Definition (…) [11]	Brescia et al.	+	+	+	−	+	−	+	−	6	Moderate

Q.1—Abstract contains the aim of the study, methods, results and conclusion

Q.2—The study provides ethic approval

Q.3—Presence of type of the study, criteria of inclusion and rejection, randomisation

Q.4—Presence of control group

Q.5—Size of the group (above 10 cases—1 point, above 20 cases—2 points)

Q.6—Statistical method stated, p-value stated

Q.7—The purpose and conclusions are clearly reported

Q.8—Limitations are reported

Among analyzed studies no late complications occurred after FESS [11, 14–16], 0–5% cases in replaceable bone lid or pedicled bone lid techniques [1, 2, 11, 15], and 15–18% in Caldwell Luc approach [16, 17]—Table 3.

**Table 3:** Rate of late complications of particular method of treatment

Author	FESS	Replaceable bone lid or pedicled bone lid	Conservative antrostomy (Caldwell-Luc)						
	Late complications	N	%	Late complications	n	%	Late complications	n	%
Matti et al. [14]	0	16	0%	–	–	–	–	–	–
Chiapasco et al. [15]	0	6	0%	0	17	0%	–	–	–
Brescia et al. [11]	0	7	0%	0	2	0%	–	–	–
Manor et al. [16]	0	1	0%	–	–	–	8*	52*	15%*
Hu et al. [2]	–	–	–	1	21	5%	–	–	–
Sgaramella et al. [1]	–	–	–	0	21	0%	–	–	–
Albu et al. [17]	–	–	–	–	–	–	9**	50**	18%**

*Lateral maxillary sinus wall antrostomy [16]

**Antrostomy through canine fossa and 'double-barrel approach'—operation through two trocars inserted via lateral wall [17]

## Discussion

Displaced foreign bodies should be always removed from maxillary sinus, to prevent sinonasal complications [1, 11, 14–16] or accidental dangerous spontaneous displacement [18].

FESS facilitates performing no intervention within the maxillary sinus wall and maintaining natural ostium drainage—resulting in low surgical trauma and low rate of complications. Nasal endoscopic techniques provide the same efficiency of foreign body removal as intraoral approaches [1] and possible inferior meatal antrostomy resolves problems with large foreign bodies and those located unfavourably to be retrieved via natural ostium [14, 19]. Another advantage is brought by the possibility of simultaneous treatment of associated sinonasal pathologies [15]. Possible presence of oroantral fistula forces to supplement FESS with an intraoral approach [11, 15].

Replaceable or pedicled bone lid techniques do invade maxillary sinus wall, which is associated with postoperative facial swelling and paresthesia [2]. However these approaches restore the integrity of the maxillary bone and, partly, Shneiderian membrane [20, 21], providing quite low rate of late complications [1, 2, 11, 15]. They bring particular application in cases related to maxillary bone involvement [11, 15].

Conservative Caldwell-Luc operation should be performed for foreign body removal only if other techniques are not available, it brings 15–18% risk of postoperative sinusitis. Frequently appear symptoms such as: facial numbness, cheek pain, cheek swelling, teeth numbness, gingival problems associated with damage of the bone and infraorbital nerve [16, 17].

Iatrogenic foreign body migration into maxillary sinus remains relatively rare complication, though exact morbidity seems to be underestimated and still rising [1, 15]. Lack of randomized trials and little investigated cases impede evaluation of methods of treatment in the previous literature. Therefore, the quality of included articles appeared rather weak—we assessed 1 paper as high quality, 3 papers as moderate quality and 3 papers as poor quality (Table 2). Those are the main limitations of our study.

## Conclusion

To conclude, FESS probably should become a gold standard for removing foreign bodies from maxillary sinus. However, there still exist the necessity for further investigation, especially comparing FESS and replaceable or pedicled bone lid techniques using prospective, randomized studies to estimate detailed outcomes.
